# Adverse events of PD-(L)1 inhibitors plus anti-VEGF(R) agents compared with PD-(L)1 inhibitors alone for cancer patients: a systematic review and meta-analysis

**DOI:** 10.3389/fphar.2023.1093194

**Published:** 2023-04-25

**Authors:** Qiyu Tang, Dawei Wu, Huiyao Huang, Hong Fang, Ying Wu, Funan Liu, Ning Li

**Affiliations:** ^1^ Clinical Trials Center, National Cancer Center/National Clinical Research Center for Cancer/Cancer Hospital, Chinese Academy of Medical Sciences and Peking Union Medical College, Beijing, China; ^2^ Phase I Clinical Trails Center, The First Hospital, China Medical University, Shenyang, China

**Keywords:** programmed cell death (ligand) 1, vascular endothelial growth factor (receptor), immune-related adverse events, treatment-related adverse events, meta-analysis

## Abstract

**Background:** Anti-PD-(L)1 antibody monotherapy or in combination with VEGF(R) blockade has been applied widely for cancer treatment. Whether combination therapy increases irAEs still remains controversial.

**Methods:** A systematic review and meta-analysis comparing PD-(L)1 and VEGF(R) blockade combination therapy with PD-(L)1 inhibitors alone was performed. Phase II or III randomized clinical trials reporting irAEs or trAEs were included. The protocol was registered with PROSPERO, CRD42021287603.

**Results:** Overall, 77 articles were included in the meta-analysis. A total of 31 studies involving 8,638 participants were pooled and an incidence for PD-(L)1 inhibitor monotherapy with any grade and grade ≥3 irAEs of 0.25 (0.20, 0.32) and 0.06 (0.05, 0.07), respectively, were reported. Two studies with 863 participants pooled for PD-(L)1 and VEGF(R) blockade showed that an incidence of any grade and grade ≥3 irAEs were 0.47 (0.30, 0.65) and 0.11 (0.08, 0.16), respectively. Regarding pairwise comparisons for irAEs, only one study was included, indicating no significant difference between the two regimens in terms of colitis, hyperthyroidism, and hypothyroidism for any grade and grade ≥3, while there was a trend of higher incidence for any grade hyperthyroidism under the combination therapy. The incidence of reactive cutaneous capillary endothelial proliferation (RCCEP) was as high as 0.80 under camrelizumab monotherapy.

**Conclusion:** Total incidences of any grade and grade ≥3 irAEs were higher in the combination treatment group. Direct comparisons indicated no significant difference between the two regimens for any grade and grade ≥3 specific irAEs. RCCEP and thyroid disorders need to be paid attention to clinically. Moreover, trials with direct comparisons are needed and the safety profiles of the two regimens should be further explored. Exploration of the mechanism of action and regulatory management of adverse events should be enhanced.

**Systematic Review Registration**: https://www.crd.york.ac.uk/prospero/display_record.php?RecordID=287603, identifier CRD42021287603

## 1 Introduction

Immune check inhibitors (ICIs) have revolutionized oncology through the approach of blocking intrinsic down-regulators of immunity and increasing antitumor immunity, as well as countering immune suppression in the tumor microenvironment (Postow et al., 2018; Brahmer et al., 2021). Programmed cell death (ligand) 1 (PD-(L)1) inhibitors monotherapy or combination regimens have been approved as first-line or second-line therapies in a range of cancer types. Anti-vascular endothelial growth factor (receptor) (VEGF(R)) agents targeting the VEGF signaling pathway, which may have synergistic effects with PD-(L)1 blockade (Meder et al., 2018; Yi et al., 2019) and potentially reverse the resistance to ICIs (Kim et al., 2019; Yi et al., 2019; Saeed et al., 2021), have been approved in combination with PD-(L)1 inhibitors for the first-line treatment of hepatocellular carcinoma (Finn et al., 2020a) and renal cell carcinoma (Motzer et al., 2020a; Powles et al., 2020a; Choueiri et al., 2021), and second-line treatment and beyond of endometrial cancer (Makker et al., 2019). In several circumstances, studies revealed that the dual combination regimen could significantly improve survival benefits compared with the PD-(L)1 inhibitor alone, representing a promising therapeutic effect (Huang et al., 2021; Chen et al., 2022).

There is accumulating evidence that ICIs are associated with immune-related adverse events (irAEs), which often demand multidisciplinary collaboration from the clinician. Presently, the mechanism of irAEs is not elucidated, which is perhaps related to the off-target effects from the excessively activated immune system as well as the production of inflammatory cytokines resulting from T-cell activation (Martins et al., 2019; Kennedy and Salama, 2020). Since trials exploring the efficacy of combination regimens are increasing, there are urgent concerns about irAEs, especially severe irAEs which are life-threatening.

A systematic review evaluating the incidence of common irAEs of single-agent PD-(L)1 inhibitor found that diarrhea (9.47%) and hypothyroidism (6.07%) were of relatively high risk (Wang et al., 2019). A similar result of hypothyroidism (5.6%) was also reported in the meta-analysis of 13 studies with 3,803 participants (Baxi et al., 2018). With respect to PD-(L)1 and VEGF(R) dual inhibitors, the risk of irAEs reported ranged broadly from 38% to 56% (Motzer et al., 2019; Powles et al., 2020a) while no meta-analysis was performed to reach a consensus. Moreover, a meta-analysis focusing on the direct comparison between PD-(L)1 blockade plus anti-VEGF(R) agents with PD-(L)1 blockade alone has not yet been conducted. Accordingly, we conducted a systematic review and meta-analysis to explore whether the incidence of irAEs increased in combination therapy, compared with anti-PD-(L)1 antibody monotherapy.

## 2 Methods

The Preferred Reporting Items for Systematic Reviews and Meta-Analyses (PRISMA) statement was applied in this report (Moher et al., 2009). The protocol was registered with the International Prospective Register of Systematic Reviews (PROSPERO), number CRD42021287603.

### 2.1 Data sources and searches

Three electronic databases (PubMed, Embase, and Cochrane CENTRAL) were systematically retrieved from inception to 22 October 2021, with language restricted to English. Keywords such as *PD-1*, *PD-L1, and randomized* were used (see Supplementary Table S1). Clinicaltrials.gov and conference proceedings (American Society of Clinical Oncology, European Society for Medical Oncology, and American Association for Cancer Research) were manually searched. References of eligible studies were also manually reviewed.

### 2.2 Selection criteria

Phase II or III randomized controlled trials that reported PD-(L)1 inhibitor monotherapy or PD-(L)1 inhibitors plus anti-VEGF(R) agents for the treatment of cancer patients irrespective of solid or hematologic malignancies were included. Trials that only compared different dosages or administration intervals were excluded. Additionally, sequential combination therapy was excluded. The primary outcomes were incidence of any grade irAEs and grade ≥3 irAEs. The secondary outcomes were incidence of any grade treatment-related adverse events (trAEs) and grade ≥3 trAEs. Adverse events were graded according to the National Cancer Institute-Common Terminology Criteria for Adverse Events (CTCAE) (CTCAE, 2017). When duplicate cohorts were reported, the most recent publication with comprehensive data was included. Two authors (TQY and WDW) first independently screened the titles and/or abstracts to identify potential trials and then checked the full-text articles for eligibility. Disagreements were resolved by a third author (LN).

### 2.3 Data extraction and risk of bias assessment

Two authors (TQY and WDW) extracted the following data using a pre-designed form independently: study characteristics (first author, publication year, NCT number, and trial name), methods (trial phase, masking status, and line of treatment), participants (cancer type, performance status, and PD-L1 expression status), interventions (intervention and comparison group regimes), and outcomes (follow-up duration, adverse event type, incidence of irAEs and trAEs (grade 1 to 5 and grade 3 to 5)). Any discrepancies were resolved by a third investigator (LN).

Two reviewers (TQY and WDW) independently evaluated the methodological quality of eligible studies using the Cochrane Collaboration’s tool based on the following items: random sequence generation, allocation concealment, blinding of participants and healthcare providers, blinding of outcome assessment, incomplete outcome data, selective outcome reporting, and other sources of bias (Higgins et al., 2011). Any conflict was resolved by a third reviewer (LN).

### 2.4 Data synthesis and statistical analyses

The effect size of the safety profile was estimated by relative risk (RR) with a corresponding 95% confidence interval (CI). Incidence of PD-(L)1 inhibitor monotherapy and in combination with anti-VEGF(R) agents were pooled, respectively, for there were few direct comparisons. In addition, pooled effects of the direct comparisons between the two regimens were also estimated using the random effects model. R software (version 3.5.3) with meta package (version 4.9–3) was used. The classic half-integer continuity correction (adding 0.5 to each cell) was used when zero adverse events were reported in any arm.

Heterogeneity was examined b*y* Cochran Q and *I* ^2^ statistic, with significance set at *p* < 0.10. *I* ^2^ of greater than 50% was considered as high risk, 25%–50% as moderate risk, and less than 25% as low risk (Higgins et al., 2003). Funnel plot and Egger’s test were employed to explore the potential publication bias and the small-study effect when more than ten studies were included. Sensitivity analysis was performed by omitting eligible studies one by one. Statistical significance was considered when *p* < 0.05 if not noted.

## 3 Results

### 3.1 Study selection

**FIGURE 1:**
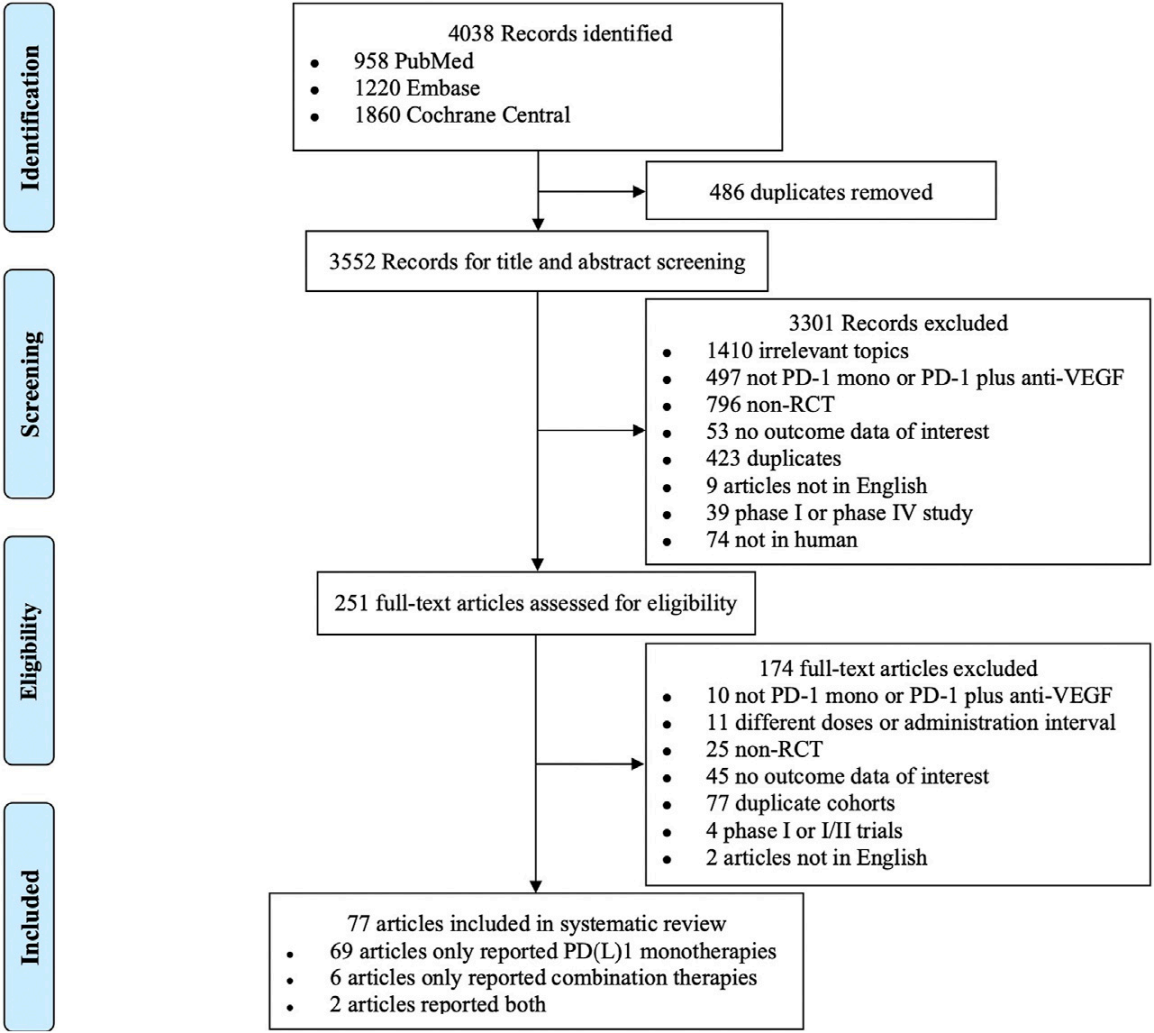
PRISMA flow chart.

A total of 4,038 records were identified, of which 486 duplicates were excluded. After screening by title and/or abstract, 251 articles were included for full-text screening. Ultimately, 77 articles [(Finn et al., 2020a; Powles et al., 2020a; Choueiri et al., 2021); (Motzer et al., 2019); (Borghaei et al., 2015; Brahmer et al., 2015; Fehrenbacher et al., 2016; Carbone et al., 2017; Hamid et al., 2017; Bang et al., 2018; D'Angelo et al., 2018; Fehrenbacher et al., 2018; Ferris et al., 2018; Larkin et al., 2018; Long et al., 2018; McDermott et al., 2018; Bonomi et al., 2019; Burtness et al., 2019; Cohen et al., 2019; Eng et al., 2019; Fradet et al., 2019; Hellmann et al., 2019; Kato et al., 2019; Larkin et al., 2019; Levy et al., 2019; Long et al., 2019; Mok et al., 2019; Necchi et al., 2019; O'Reilly et al., 2019; Pujol et al., 2019; Rini et al., 2019; Robert et al., 2019; Scherpereel et al., 2019; Siu et al., 2019; Theelen et al., 2019; Andre et al., 2020; Ferris et al., 2020; Finn et al., 2020b; Galsky et al., 2020; Herbst et al., 2020; Huang et al., 2020; Kojima et al., 2020; Motzer et al., 2020b; Planchard et al., 2020; Popat et al., 2020; Powles et al., 2020b; Reardon et al., 2020; Rizvi et al., 2020; Robert et al., 2020; Shitara et al., 2020; Zamarin et al., 2020; Zhang et al., 2020; Boku et al., 2021; Boyer et al., 2021; Gettinger et al., 2021; Gogas et al., 2021; Hamanishi et al., 2021; Heudobler et al., 2021; Jassem et al., 2021; Kuruvilla et al., 2021; Liu et al., 2021; Lu et al., 2021; Mahmood et al., 2021; McBride et al., 2021; Motzer et al., 2021; Nayak et al., 2021; Park et al., 2021; Powles et al., 2021; Pujade-Lauraine et al., 2021; Reck et al., 2021; Sezer et al., 2021; Spigel et al., 2021; van der Heijden et al., 2021; Winer et al., 2021; Chawla et al., 2022)] were included in the meta-analysis. The most frequent reasons for exclusion during full-text screening were duplicate cohorts (77 records) and no outcome data of interest (45 records). (See Figure 1).

### 3.2 Basic characteristics

Among 77 trials included, 54 (70.1%) studies [(Finn et al., 2020a; Powles et al., 2020a; Choueiri et al., 2021); (Motzer et al., 2019); (Borghaei et al., 2015); (Brahmer et al., 2015); (Carbone et al., 2017); (Bang et al., 2018); (Fehrenbacher et al., 2018; Ferris et al., 2018; Larkin et al., 2018); (Burtness et al., 2019; Cohen et al., 2019; Eng et al., 2019; Fradet et al., 2019; Hellmann et al., 2019; Kato et al., 2019; Larkin et al., 2019); (Long et al., 2019); (Mok et al., 2019); (Rini et al., 2019); (Robert et al., 2019); (Andre et al., 2020; Finn et al., 2020b; Ferris et al., 2020; Galsky et al., 2020); (Motzer et al., 2020b; Powles et al., 2020b; Huang et al., 2020; Kojima et al., 2020; Planchard et al., 2020; Popat et al., 2020; Reardon et al., 2020; Rizvi et al., 2020; Robert et al., 2020; Shitara et al., 2020); (Boku et al., 2021; Boyer et al., 2021; Gettinger et al., 2021; Gogas et al., 2021; Hamanishi et al., 2021); (Jassem et al., 2021); (Kuruvilla et al., 2021); (Lu et al., 2021); (Motzer et al., 2021); (Park et al., 2021; Powles et al., 2021; Pujade-Lauraine et al., 2021; Reck et al., 2021; Sezer et al., 2021; Spigel et al., 2021; van der Heijden et al., 2021; Winer et al., 2021); (Fuchs et al., 2022)] were phase III trials, 22 (28.6%) (Fehrenbacher et al., 2016; Hamid et al., 2017; D'Angelo et al., 2018; Long et al., 2018; McDermott et al., 2018; Bonomi et al., 2019; Levy et al., 2019; Necchi et al., 2019; O'Reilly et al., 2019; Pujol et al., 2019; Scherpereel et al., 2019; Siu et al., 2019; Theelen et al., 2019; Zamarin et al., 2020; Zhang et al., 2020; Heudobler et al., 2021; Liu et al., 2021; Mahmood et al., 2021; McBride et al., 2021; Nayak et al., 2021; Chawla et al., 2022; Singh et al., 2022) were phase II, and 1 (1.3%) (Herbst et al., 2020) was phase II/III trial. Regimens in 29 (37.7%) studies (Carbone et al., 2017; Hamid et al., 2017; Long et al., 2018; McDermott et al., 2018; Burtness et al., 2019; Hellmann et al., 2019; Larkin et al., 2019; Long et al., 2019; Mok et al., 2019; Motzer et al., 2019; Rini et al., 2019; Finn et al., 2020a; Andre et al., 2020; Powles et al., 2020a; Powles et al., 2020b; Galsky et al., 2020; Rizvi et al., 2020; Robert et al., 2020; Shitara et al., 2020; Boyer et al., 2021; Choueiri et al., 2021; Gogas et al., 2021; Jassem et al., 2021; Mahmood et al., 2021; McBride et al., 2021; Motzer et al., 2021; Powles et al., 2021; Reck et al., 2021; Sezer et al., 2021) were frontline (1 or ≥1 line) therapies and in 32 (41.6%) studies (Brahmer et al., 2015; D'Angelo et al., 2018; Ferris et al., 2018; Larkin et al., 2018; Bonomi et al., 2019; Cohen et al., 2019; Fradet et al., 2019; Kato et al., 2019; Levy et al., 2019; O'Reilly et al., 2019; Pujol et al., 2019; Siu et al., 2019; Theelen et al., 2019; Ferris et al., 2020; Finn et al., 2020b; Herbst et al., 2020; Huang et al., 2020; Kojima et al., 2020; Popat et al., 2020; Reardon et al., 2020; Zhang et al., 2020; Gettinger et al., 2021; Hamanishi et al., 2021; Heudobler et al., 2021; Kuruvilla et al., 2021; Lu et al., 2021; Park et al., 2021; Spigel et al., 2021; van der Heijden et al., 2021; Chawla et al., 2022; Fuchs et al., 2022; Singh et al., 2022) were second-line (2 or ≥2 line) and in 6 (7.8%) studies (Bang et al., 2018; Eng et al., 2019; Necchi et al., 2019; Planchard et al., 2020; Boku et al., 2021; Liu et al., 2021) were third-line setting (3 or ≥3 line). In addition, one (1.3%) study (Robert et al., 2019) focused on first/second-line therapy, seven (9.1%) studies (Borghaei et al., 2015; Fehrenbacher et al., 2016; Fehrenbacher et al., 2018; Scherpereel et al., 2019; Motzer et al., 2020b; Nayak et al., 2021; Winer et al., 2021) on second/third-line, and two (2.6%) studies (Zamarin et al., 2020; Pujade-Lauraine et al., 2021) on second to fourth line therapy. PD-L1 expression status in 54 (70.1%) studies was noted as unselected [(Winer et al., 2021; Chawla et al., 2022; Fuchs et al., 2022; Singh et al., 2022); (Winer et al., 2021; Chawla et al., 2022; Fuchs et al., 2022; Singh et al., 2022); (Winer et al., 2021; Chawla et al., 2022; Fuchs et al., 2022; Singh et al., 2022); (Borghaei et al., 2015; Brahmer et al., 2015; Fehrenbacher et al., 2016); (Borghaei et al., 2015; Brahmer et al., 2015; Fehrenbacher et al., 2016); (Borghaei et al., 2015; Brahmer et al., 2015; Fehrenbacher et al., 2016); (Fehrenbacher et al., 2018; Ferris et al., 2018; Larkin et al., 2018; Long et al., 2018; McDermott et al., 2018); (Burtness et al., 2019; Cohen et al., 2019; Eng et al., 2019; Fradet et al., 2019); (Kato et al., 2019; Larkin et al., 2019; Levy et al., 2019; Long et al., 2019); (O'Reilly et al., 2019; Pujol et al., 2019; Rini et al., 2019; Robert et al., 2019; Scherpereel et al., 2019); (Theelen et al., 2019); (Ferris et al., 2020); (Galsky et al., 2020); (Motzer et al., 2020b; Huang et al., 2020; Kojima et al., 2020); (Powles et al., 2020b; Popat et al., 2020; Reardon et al., 2020; Rizvi et al., 2020; Robert et al., 2020); (Zamarin et al., 2020); (Boku et al., 2021); (Gettinger et al., 2021; Gogas et al., 2021; Hamanishi et al., 2021); (Kuruvilla et al., 2021; Liu et al., 2021; Lu et al., 2021); (McBride et al., 2021; Motzer et al., 2021; Nayak et al., 2021; Park et al., 2021; Powles et al., 2021; Pujade-Lauraine et al., 2021); (Spigel et al., 2021; van der Heijden et al., 2021; Winer et al., 2021)]. There were 59 (76.6%) trials [(Finn et al., 2020a); (Motzer et al., 2019); (Borghaei et al., 2015; Brahmer et al., 2015; Fehrenbacher et al., 2016; Carbone et al., 2017; Hamid et al., 2017; Bang et al., 2018; D'Angelo et al., 2018; Fehrenbacher et al., 2018; Ferris et al., 2018; Larkin et al., 2018); (Burtness et al., 2019; Cohen et al., 2019; Eng et al., 2019); (Hellmann et al., 2019; Kato et al., 2019; Larkin et al., 2019; Levy et al., 2019; Long et al., 2019; Mok et al., 2019; Necchi et al., 2019; O'Reilly et al., 2019); (Robert et al., 2019; Scherpereel et al., 2019; Siu et al., 2019; Theelen et al., 2019; Andre et al., 2020; Finn et al., 2020b; Ferris et al., 2020); (Herbst et al., 2020; Huang et al., 2020; Kojima et al., 2020); (Powles et al., 2020b; Planchard et al., 2020; Popat et al., 2020); (Rizvi et al., 2020; Robert et al., 2020; Shitara et al., 2020); (Zhang et al., 2020; Boku et al., 2021; Boyer et al., 2021); (Gogas et al., 2021; Hamanishi et al., 2021; Heudobler et al., 2021; Jassem et al., 2021; Kuruvilla et al., 2021; Liu et al., 2021; Lu et al., 2021; Mahmood et al., 2021); (Park et al., 2021); (Pujade-Lauraine et al., 2021; Reck et al., 2021; Sezer et al., 2021; Spigel et al., 2021); (Winer et al., 2021; Chawla et al., 2022; Fuchs et al., 2022; Singh et al., 2022)] with Eastern Cooperative Oncology Group (ECOG) performance status score of 0–1, 7 trials (9.1%) (Long et al., 2018; Fradet et al., 2019; Pujol et al., 2019; Galsky et al., 2020; Zamarin et al., 2020; McBride et al., 2021; Powles et al., 2021) with ECOG score of 0–2, 1 trial (1.3%) (Bonomi et al., 2019) with ECOG score of 2–3, 8 (10.4%) studies (McDermott et al., 2018; Rini et al., 2019; Powles et al., 2020a; Motzer et al., 2020b; Reardon et al., 2020; Choueiri et al., 2021; Motzer et al., 2021; Nayak et al., 2021) with Karnofsky performance score ≥70 and 1 (1.3%) study (Gettinger et al., 2021) with Zubrod performance status score of 0–1. As for dual combination therapy, six regimens were reported in our study: pembrolizumab plus bevacizumab (Nayak et al., 2021), pembrolizumab plus lenvatinib (Motzer et al., 2021), pembrolizumab plus axitinib (Powles et al., 2020a), nivolumab plus cabozantinib (Choueiri et al., 2021), atezolizumab plus bevacizumab (McDermott et al., 2018; Rini et al., 2019; Finn et al., 2020a), avelumab plus axitinib (Motzer et al., 2019). (See Supplementary Table S2).

### 3.3 Risk of bias

Bias of 71 (92.2%) studies [(Finn et al., 2020a; Powles et al., 2020a; Choueiri et al., 2021); (Motzer et al., 2019); (Borghaei et al., 2015; Brahmer et al., 2015; Fehrenbacher et al., 2016; Carbone et al., 2017; Hamid et al., 2017; Bang et al., 2018; D'Angelo et al., 2018; Fehrenbacher et al., 2018; Ferris et al., 2018; Larkin et al., 2018; Long et al., 2018; McDermott et al., 2018; Bonomi et al., 2019; Burtness et al., 2019; Cohen et al., 2019; Eng et al., 2019; Fradet et al., 2019; Hellmann et al., 2019; Kato et al., 2019); (Mok et al., 2019; Necchi et al., 2019; O'Reilly et al., 2019; Pujol et al., 2019; Rini et al., 2019; Robert et al., 2019; Scherpereel et al., 2019; Siu et al., 2019; Theelen et al., 2019; Andre et al., 2020; Ferris et al., 2020); (Motzer et al., 2020b; Powles et al., 2020b; Galsky et al., 2020; Herbst et al., 2020; Huang et al., 2020; Kojima et al., 2020; Planchard et al., 2020; Popat et al., 2020; Reardon et al., 2020; Rizvi et al., 2020; Robert et al., 2020; Shitara et al., 2020; Zamarin et al., 2020; Zhang et al., 2020); (Gettinger et al., 2021; Gogas et al., 2021; Hamanishi et al., 2021; Heudobler et al., 2021; Jassem et al., 2021; Kuruvilla et al., 2021; Liu et al., 2021; Lu et al., 2021; Mahmood et al., 2021; McBride et al., 2021; Motzer et al., 2021; Nayak et al., 2021; Park et al., 2021; Powles et al., 2021; Pujade-Lauraine et al., 2021; Reck et al., 2021; Sezer et al., 2021; Spigel et al., 2021; van der Heijden et al., 2021; Winer et al., 2021; Chawla et al., 2022; Fuchs et al., 2022; Singh et al., 2022)] were regarded as high risk and 6 (7.8%) (Larkin et al., 2019; Levy et al., 2019; Long et al., 2019; Finn et al., 2020b; Boku et al., 2021; Boyer et al., 2021) were low risk. 67 (87.0%) studies [(Finn et al., 2020a; Powles et al., 2020a; Choueiri et al., 2021); (Motzer et al., 2019); (Borghaei et al., 2015; Brahmer et al., 2015; Fehrenbacher et al., 2016; Carbone et al., 2017); (Bang et al., 2018; D'Angelo et al., 2018; Fehrenbacher et al., 2018; Ferris et al., 2018; Larkin et al., 2018; Long et al., 2018; McDermott et al., 2018; Bonomi et al., 2019; Burtness et al., 2019; Cohen et al., 2019; Eng et al., 2019; Fradet et al., 2019; Hellmann et al., 2019; Kato et al., 2019); (Mok et al., 2019; Necchi et al., 2019; O'Reilly et al., 2019; Pujol et al., 2019; Rini et al., 2019; Robert et al., 2019; Scherpereel et al., 2019; Siu et al., 2019; Theelen et al., 2019; Andre et al., 2020; Ferris et al., 2020); (Motzer et al., 2020b; Powles et al., 2020b; Herbst et al., 2020; Huang et al., 2020; Kojima et al., 2020; Planchard et al., 2020; Popat et al., 2020; Reardon et al., 2020; Rizvi et al., 2020); (Zamarin et al., 2020); (Zhang et al., 2020); (Gettinger et al., 2021; Gogas et al., 2021; Hamanishi et al., 2021; Heudobler et al., 2021; Jassem et al., 2021; Kuruvilla et al., 2021; Liu et al., 2021; Lu et al., 2021; Mahmood et al., 2021; McBride et al., 2021; Motzer et al., 2021; Nayak et al., 2021; Park et al., 2021; Powles et al., 2021; Pujade-Lauraine et al., 2021; Reck et al., 2021; Sezer et al., 2021; Spigel et al., 2021; van der Heijden et al., 2021; Winer et al., 2021; Chawla et al., 2022; Fuchs et al., 2022; Singh et al., 2022)] were open-label and 2 (2.6%) studies (Hamid et al., 2017; Shitara et al., 2020) were partially blinded (patients and investigators masked only to the pembrolizumab dose in one study and patients masked only to the combination therapy groups and unblinded to pembrolizumab monotherapy in the other study), which was regarded as the most common reason for high risk. Only 8 (10.4%) trials (Larkin et al., 2019; Levy et al., 2019; Long et al., 2019; Finn et al., 2020b; Galsky et al., 2020; Robert et al., 2020; Boku et al., 2021; Boyer et al., 2021) were set as double-blind or quadruple-blind. (See Supplementary Table S3).

### 3.4 Safety assessment

#### 3.4.1 irAEs

**FIGURE 2:**
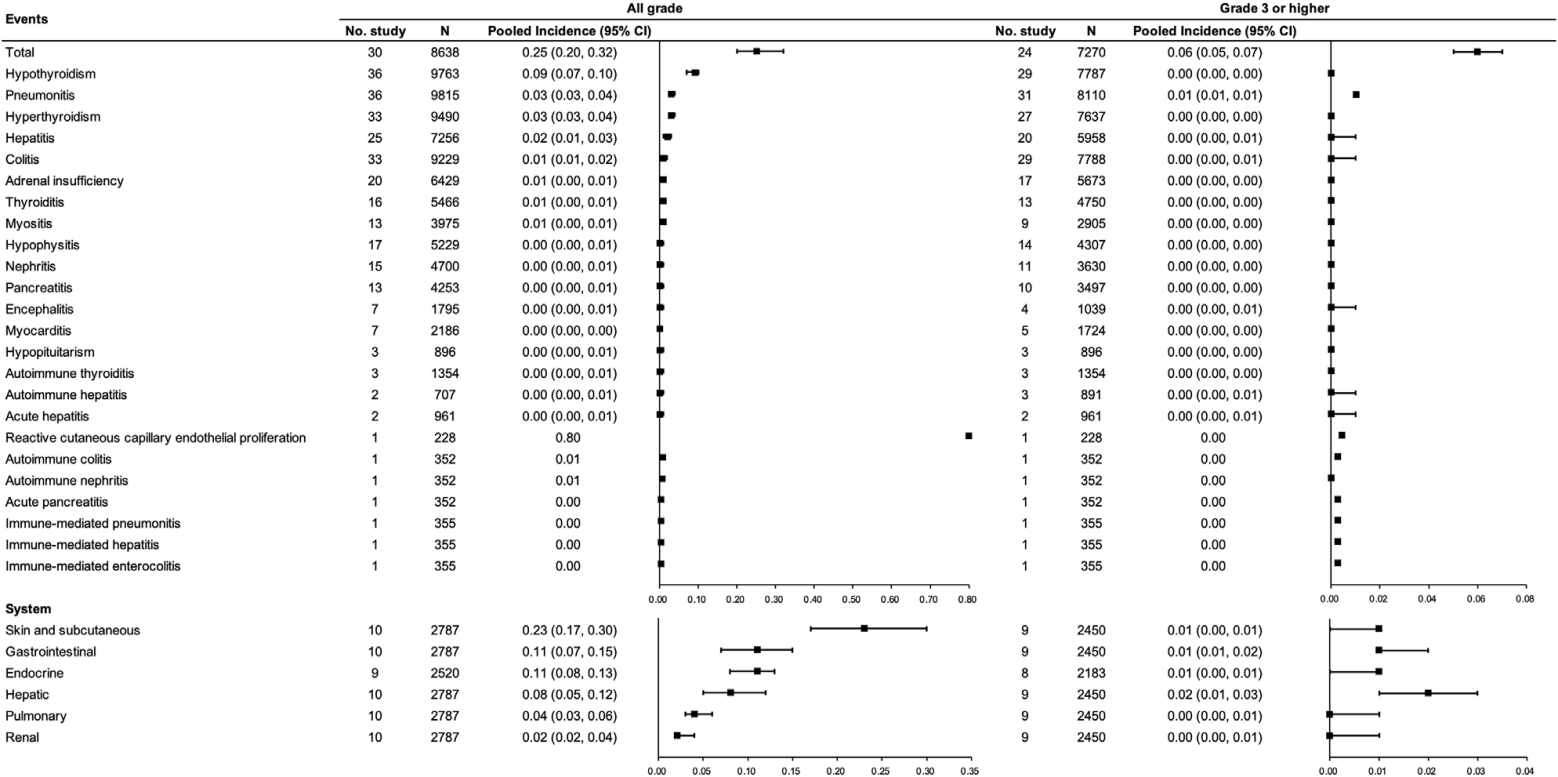
Incidence of irAEs for PD-(L)1 inhibitor monotherapy. Note: an incidence of 0.00 indicates a rate <0.005.

For PD-(L)1 inhibitor monotherapy, 49 studies [(Borghaei et al., 2015; Brahmer et al., 2015; Fehrenbacher et al., 2016; Carbone et al., 2017; Hamid et al., 2017; Bang et al., 2018); (Fehrenbacher et al., 2018; Ferris et al., 2018; Larkin et al., 2018); (Cohen et al., 2019); (Fradet et al., 2019); (Hellmann et al., 2019); (Larkin et al., 2019); (Long et al., 2019); (Mok et al., 2019); (Pujol et al., 2019); (Robert et al., 2019; Scherpereel et al., 2019; Siu et al., 2019; Theelen et al., 2019; Andre et al., 2020); (Finn et al., 2020b; Motzer et al., 2020b; Galsky et al., 2020; Herbst et al., 2020; Huang et al., 2020; Kojima et al., 2020); (Powles et al., 2020b; Popat et al., 2020; Reardon et al., 2020; Rizvi et al., 2020); (Shitara et al., 2020); (Boyer et al., 2021); (Gettinger et al., 2021); (Hamanishi et al., 2021); (Jassem et al., 2021; Kuruvilla et al., 2021; Liu et al., 2021; Lu et al., 2021); (Nayak et al., 2021; Park et al., 2021; Powles et al., 2021; Pujade-Lauraine et al., 2021; Reck et al., 2021; Sezer et al., 2021; Spigel et al., 2021; van der Heijden et al., 2021; Winer et al., 2021); (Fuchs et al., 2022)] with 13,206 participants were included, among which 30 studies (Hamid et al., 2017; Bang et al., 2018; Fehrenbacher et al., 2018; Cohen et al., 2019; Fradet et al., 2019; Mok et al., 2019; Robert et al., 2019; Siu et al., 2019; Theelen et al., 2019; Andre et al., 2020; Finn et al., 2020b; Powles et al., 2020b; Galsky et al., 2020; Herbst et al., 2020; Huang et al., 2020; Kojima et al., 2020; Rizvi et al., 2020; Shitara et al., 2020; Boyer et al., 2021; Gettinger et al., 2021; Hamanishi et al., 2021; Jassem et al., 2021; Kuruvilla et al., 2021; Liu et al., 2021; Park et al., 2021; Pujade-Lauraine et al., 2021; Reck et al., 2021; Sezer et al., 2021; Winer et al., 2021; Fuchs et al., 2022) involving 8,638 participants were included in the pooled analysis of the total incidence of irAEs. Incidences of any grade and grade ≥3 irAEs for PD-(L)1 inhibitors were 0.25 (95% CI 0.20–0.32) and 0.06 (95% CI 0.05–0.07), respectively. The most common any grade irAEs were skin and subcutaneous, gastrointestinal, and endocrine (>10%). Grade ≥3 hepatic irAE was more frequent than other sites (0.02, 95% CI 0.01–0.03). Regarding the concrete type of any grade irAEs, reactive cutaneous capillary endothelial proliferation (RCCEP), which was reported in one study, occurred in up to 80% of the participants though most of them were assessed as grade 1 to 2. Apart from that, hypothyroidism, pneumonitis, hyperthyroidism, and hepatitis were relatively frequent (≥2%). Concerning grade ≥3 irAEs, grade ≥3 pneumonitis was more frequent. (See Figure 2).

**FIGURE 3:**
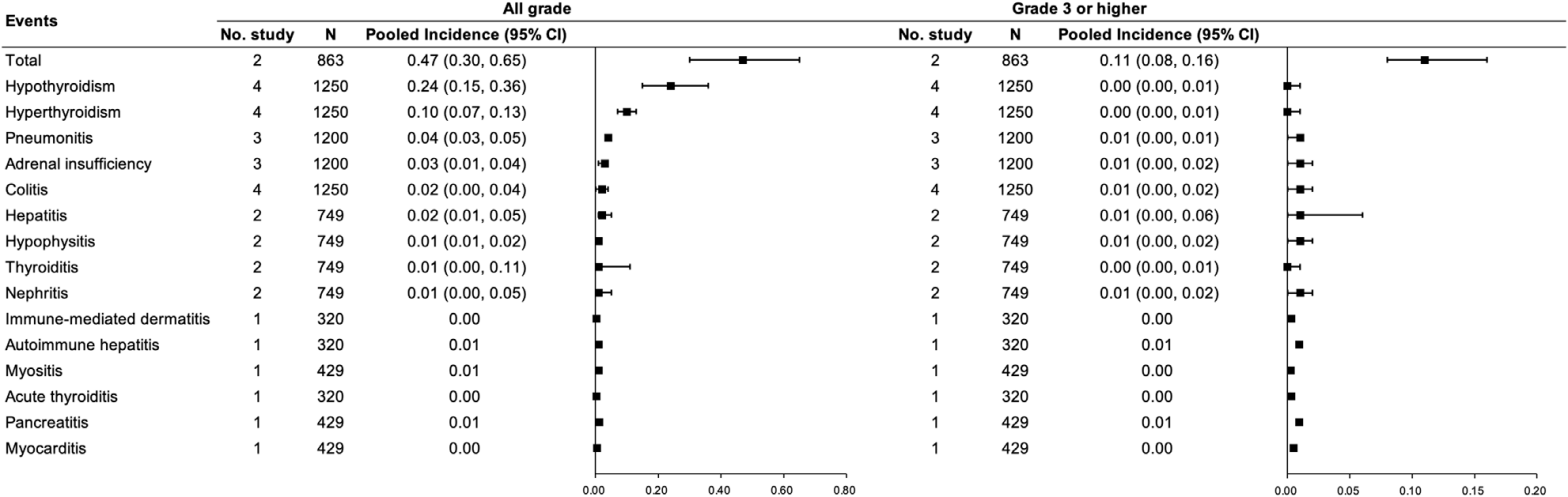
Incidence of irAEs for PD-(L)1 inhibitors combined with anti-VEGF(R) agents therapy. Note: an incidence of 0.00 indicates a rate <0.005.

Regarding the combination therapy, five studies (Motzer et al., 2019; Rini et al., 2019; Powles et al., 2020a; Choueiri et al., 2021; Nayak et al., 2021) with 1,684 participants were included, of which two studies (Motzer et al., 2019; Powles et al., 2020a) with 863 participants were included in the pooled analysis of the total incidence of any grade irAEs for PD-(L)1 inhibitors and anti-VEGF(R) agents dual combination therapy. Incidences of any grade and grade ≥3 irAEs were 0.47 (95% CI 0.30–0.65) and 0.11 (95% CI 0.08–0.16), respectively. The most common any grade irAEs were hypothyroidism and hyperthyroidism (≥10%) (See Figure 3).

**FIGURE 4:**
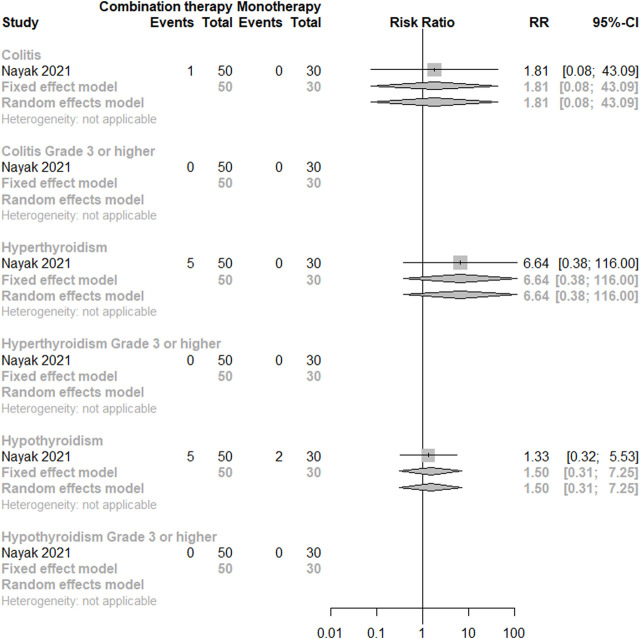
Direct comparisons of PD-(L)1 inhibitor monotherapy and in combination with anti-VEGF(R) agents therapy for irAEs.

As for direct comparisons for irAEs, only one study was included (Nayak et al., 2021), indicating no significant difference between the monotherapy and the dual combination regimen in terms of colitis, hyperthyroidism, and hypothyroidism for any grade and grade 3 or higher. There is a trend of higher incidence for any grade hyperthyroidism under combination therapy. (See Figure 4).

#### 3.4.2 trAEs

**FIGURE 5:**
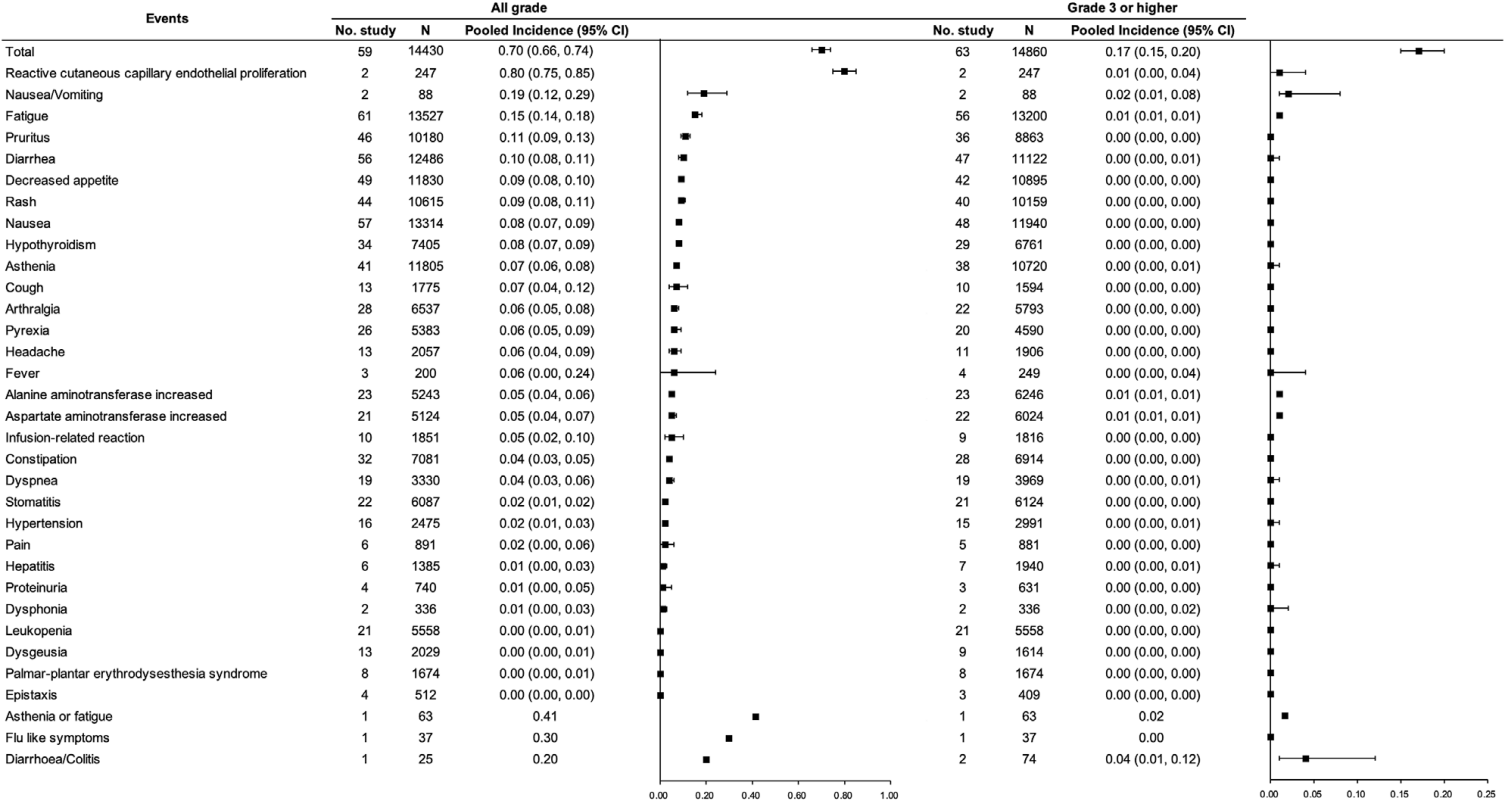
Incidence of trAEs for PD-(L)1 inhibitor monotherapy. Note: an incidence of 0.00 indicates a rate <0.005.

A total of 71 studies [(Borghaei et al., 2015; Brahmer et al., 2015; Fehrenbacher et al., 2016; Carbone et al., 2017; Hamid et al., 2017; Bang et al., 2018; D'Angelo et al., 2018; Fehrenbacher et al., 2018; Ferris et al., 2018; Larkin et al., 2018; Long et al., 2018; McDermott et al., 2018; Bonomi et al., 2019; Burtness et al., 2019; Cohen et al., 2019; Eng et al., 2019; Fradet et al., 2019; Hellmann et al., 2019; Kato et al., 2019; Larkin et al., 2019; Levy et al., 2019; Long et al., 2019; Mok et al., 2019; Necchi et al., 2019; O'Reilly et al., 2019; Pujol et al., 2019); (Robert et al., 2019; Scherpereel et al., 2019; Siu et al., 2019; Theelen et al., 2019; Andre et al., 2020; Finn et al., 2020b; Motzer et al., 2020b; Powles et al., 2020b; Ferris et al., 2020; Galsky et al., 2020; Herbst et al., 2020; Huang et al., 2020; Kojima et al., 2020; Planchard et al., 2020; Popat et al., 2020; Reardon et al., 2020; Rizvi et al., 2020; Robert et al., 2020; Shitara et al., 2020; Zamarin et al., 2020; Zhang et al., 2020; Boku et al., 2021; Boyer et al., 2021; Gettinger et al., 2021; Gogas et al., 2021; Hamanishi et al., 2021; Heudobler et al., 2021; Jassem et al., 2021; Kuruvilla et al., 2021; Liu et al., 2021; Lu et al., 2021; Mahmood et al., 2021; McBride et al., 2021); (Nayak et al., 2021; Park et al., 2021; Powles et al., 2021; Pujade-Lauraine et al., 2021; Reck et al., 2021; Sezer et al., 2021; Spigel et al., 2021; van der Heijden et al., 2021; Winer et al., 2021; Chawla et al., 2022; Fuchs et al., 2022; Singh et al., 2022)] with 15,465 participants were included, among which 59 studies [(Borghaei et al., 2015; Brahmer et al., 2015; Fehrenbacher et al., 2016; Carbone et al., 2017; Hamid et al., 2017; Bang et al., 2018); (Fehrenbacher et al., 2018; Ferris et al., 2018; Larkin et al., 2018; Long et al., 2018; McDermott et al., 2018); (Burtness et al., 2019; Cohen et al., 2019; Eng et al., 2019; Fradet et al., 2019; Hellmann et al., 2019; Kato et al., 2019; Larkin et al., 2019; Levy et al., 2019; Long et al., 2019; Mok et al., 2019); (O'Reilly et al., 2019); (Robert et al., 2019; Scherpereel et al., 2019; Siu et al., 2019); (Andre et al., 2020; Finn et al., 2020b; Motzer et al., 2020b; Powles et al., 2020b; Ferris et al., 2020; Galsky et al., 2020; Herbst et al., 2020; Huang et al., 2020; Kojima et al., 2020; Planchard et al., 2020; Popat et al., 2020; Reardon et al., 2020; Rizvi et al., 2020; Robert et al., 2020; Shitara et al., 2020); (Zhang et al., 2020); (Boyer et al., 2021); (Gogas et al., 2021; Hamanishi et al., 2021; Heudobler et al., 2021; Jassem et al., 2021; Kuruvilla et al., 2021; Liu et al., 2021; Lu et al., 2021); (McBride et al., 2021); (Park et al., 2021; Powles et al., 2021; Pujade-Lauraine et al., 2021; Reck et al., 2021; Sezer et al., 2021; Spigel et al., 2021; van der Heijden et al., 2021); (Chawla et al., 2022); (Fuchs et al., 2022)] involving 14,430 participants and 63 studies [(Borghaei et al., 2015; Brahmer et al., 2015; Fehrenbacher et al., 2016; Carbone et al., 2017; Hamid et al., 2017; Bang et al., 2018; D'Angelo et al., 2018; Fehrenbacher et al., 2018; Ferris et al., 2018; Larkin et al., 2018; Long et al., 2018); (Bonomi et al., 2019; Burtness et al., 2019; Cohen et al., 2019; Eng et al., 2019; Fradet et al., 2019; Hellmann et al., 2019; Kato et al., 2019; Larkin et al., 2019; Levy et al., 2019; Long et al., 2019; Mok et al., 2019); (O'Reilly et al., 2019); (Robert et al., 2019; Scherpereel et al., 2019; Siu et al., 2019); (Andre et al., 2020; Finn et al., 2020b; Motzer et al., 2020b; Powles et al., 2020b; Ferris et al., 2020; Galsky et al., 2020; Herbst et al., 2020; Huang et al., 2020; Kojima et al., 2020; Planchard et al., 2020; Popat et al., 2020; Reardon et al., 2020; Rizvi et al., 2020; Robert et al., 2020; Shitara et al., 2020; Zamarin et al., 2020; Zhang et al., 2020); (Boyer et al., 2021; Gettinger et al., 2021; Gogas et al., 2021; Hamanishi et al., 2021; Heudobler et al., 2021; Jassem et al., 2021; Kuruvilla et al., 2021; Liu et al., 2021; Lu et al., 2021); (McBride et al., 2021); (Park et al., 2021; Powles et al., 2021; Pujade-Lauraine et al., 2021; Reck et al., 2021; Sezer et al., 2021; Spigel et al., 2021; van der Heijden et al., 2021; Winer et al., 2021; Chawla et al., 2022; Fuchs et al., 2022)] with 14,860 participants were included for the pooled analysis of the incidence of any grade trAEs and grade ≥3 trAEs for PD-(L)1 inhibitor monotherapy, respectively. Incidences of any grade and grade ≥3 trAEs were 0.70 (95% CI 0.66–0.74) and 0.17 (95% CI 0.15–0.20), respectively. The most frequent (≥10%) any grade trAEs were reactive cutaneous capillary endothelial proliferation (RCCEP), nausea/vomiting, fatigue, pruritus, and diarrhea in the pooled analysis. In particular, RCCEP had an extremely high risk of 0.80 (0.75, 0.85), though most were assessed as grades 1 to 2. (See Figure 5).

**FIGURE 6:**
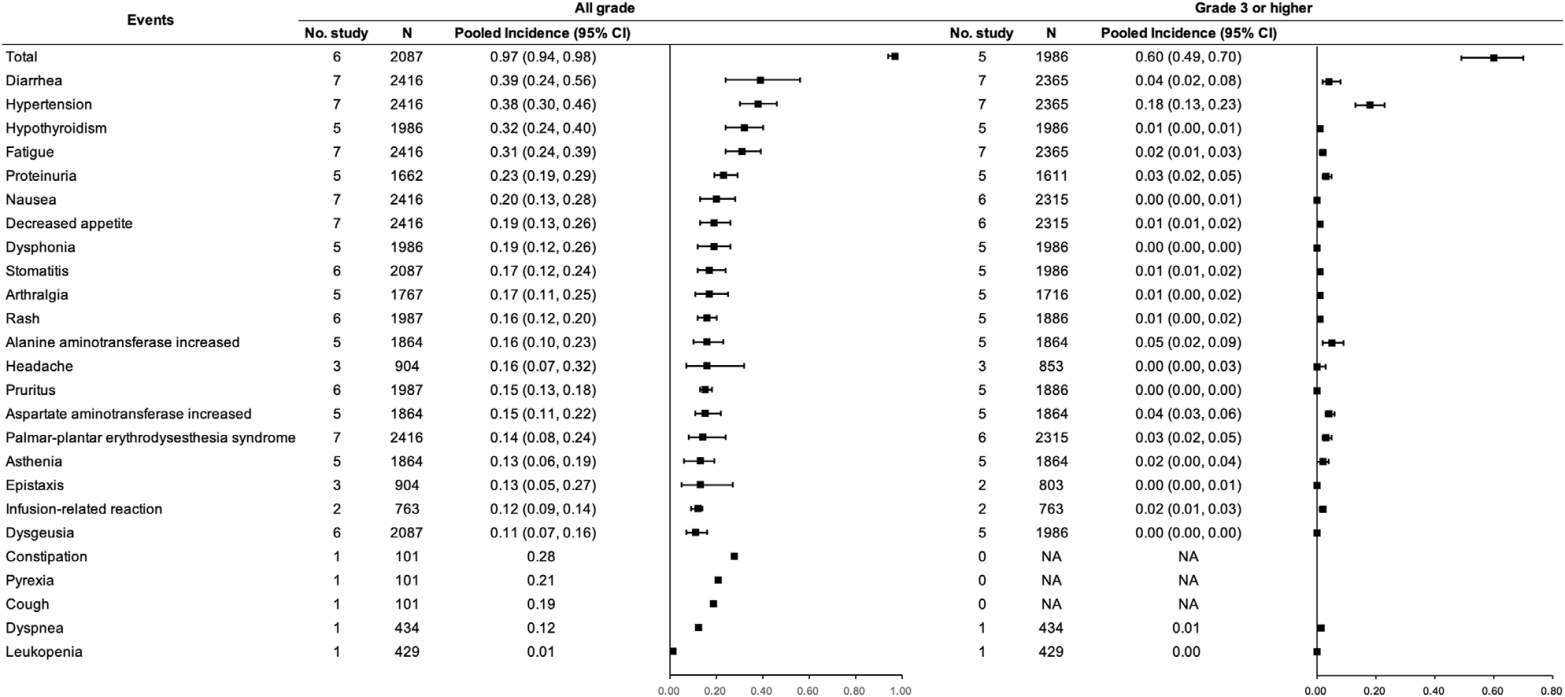
Incidence of trAEs for PD-(L)1 inhibitors combined with anti-VEGF(R) agents therapy. Note: An incidence of 0.00 indicates a rate <0.005.

With respect to the combination therapy, eight studies (McDermott et al., 2018; Motzer et al., 2019; Rini et al., 2019; Finn et al., 2020a; Powles et al., 2020a; Choueiri et al., 2021; Motzer et al., 2021; Nayak et al., 2021) with 2,466 participants were included, among which six studies (McDermott et al., 2018; Motzer et al., 2019; Rini et al., 2019; Powles et al., 2020a; Choueiri et al., 2021; Motzer et al., 2021) involving 2087 participants were included for the pooled analysis of the incidence of trAEs for PD-(L)1 inhibitors and anti-VEGF(R) agents dual combination therapy. Incidences of any grade and grade ≥3 trAEs were 0.97 (95% CI 0.94–0.98) and 0.60 (95% CI 0.49–0.70), respectively. Any grade diarrhea, hypertension, hypothyroidism, fatigue, proteinuria, and nausea were frequent (≥20%). In addition, constipation and pyrexia reported in only one study were with an incidence of more than 0.20 (McDermott et al., 2018). Regarding grade ≥3 trAEs, hypertension was the most frequent (>10%). (See Figure 6).

**FIGURE 7:**
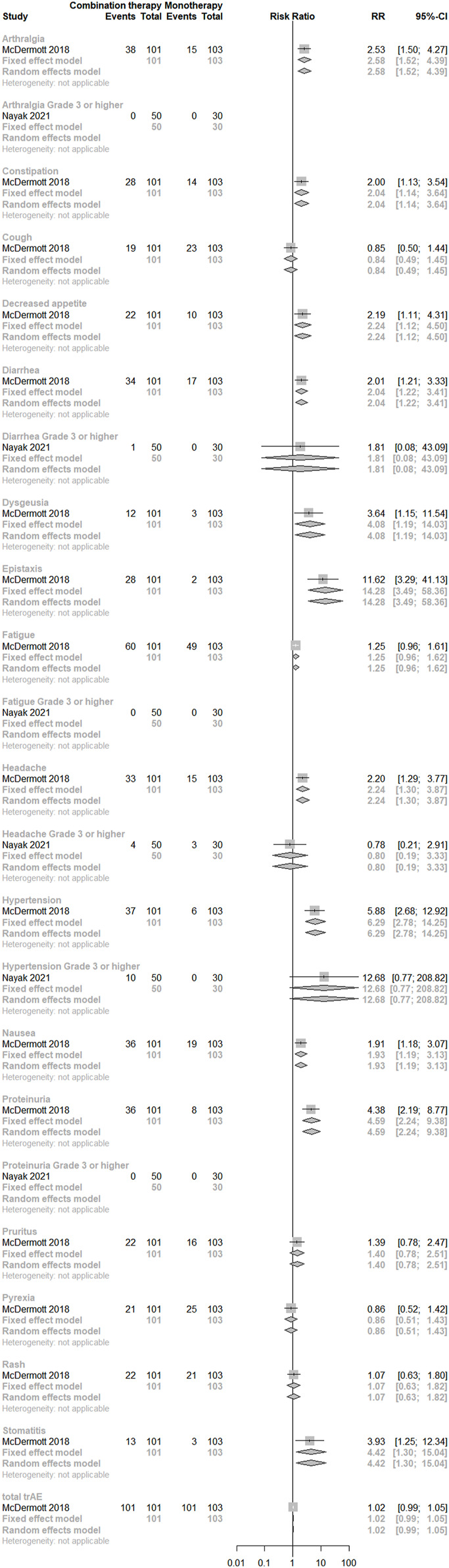
Direct comparisons of PD-(L)1 inhibitor monotherapy and in combination with anti-VEGF(R) agents therapy for trAEs.

Concerning direct comparisons for trAEs, two studies (McDermott et al., 2018; Nayak et al., 2021) were included and no significant difference was observed between the two regimens. However, risks of gastrointestinal disorders (constipation, nausea, diarrhea, and stomatitis), metabolism and nutrition disorders (decreased appetite), musculoskeletal and connective tissue disorders (arthralgia), nervous system disorders (dysgeusia and headache), respiratory, thoracic, and mediastinal disorders (epistaxis), vascular disorders (hypertension), and renal and urinary disorders (proteinuria) were significantly higher in the combination treatment group than in the monotherapy group (*p* < 0.05). (See Figure 7).

## 4 Discussion

### 4.1 Summary of evidence

We completed a systematic review to comprehensively summarize the incidence of irAEs and trAEs with PD-(L)1 inhibitor alone and in combination with VEGF(R) blockades. This study was the first systematic review comparing the safety of the two regimens directly. Our meta-analysis demonstrated that the incidence of irAEs was 0.25 (95% CI 0.20–0.32) for PD-(L)1 inhibitor monotherapy and 0.47 (95% CI 0.30–0.65) for PD-(L)1 and VEGF(R) inhibitor combination therapy, which were usually underestimated. Sensitivity analyses did not reveal essential changes in pooled results.

A preclinical study illustrated that dual blockades of PD-1 and VEGFR-2 could promote vascular normalization and enhance antitumor immune responses (Shigeta et al., 2020). Simultaneously, some irAEs tended to increase. For example, the risk of thyroid disorders, although no significant difference was observed in the head-to-head comparison (N = 80), appeared to be higher under combination therapy in the indirect comparison (N > 1,000), which may require the clinician’s attention. Moreover, several VEGF(R) blockades also have an influence on the thyroid (Ivy et al., 2009), which may affect the judgment on the relevance between irAE and PD-(L)1 inhibitors. However, according to the guideline of the Center for Drug Evaluation in China, it is also judged as an irAE conservatively, which may lead to an increase in the proportion of such toxicities. Therefore, thyroid hormone levels should be regularly monitored. Additionally, our study displayed that pneumonitis and myocarditis, which were serious and were deteriorating patients’ prognosis and quality of life were of similar risks between the two groups. What’s more, the incidence of grade ≥3 hepatic irAE was higher for PD-1 inhibitor monotherapy than combination therapy in our study (0.02 vs. 0.01). It is likely that the incidence of grade ≥3 hepatic irAE (0.02, 95% CI 0.01–0.03) referred to the hepatic system including several subtypes of adverse events, while the combination therapy group only reported hepatitis and autoimmune hepatitis with hepatic system risk not mentioned.

IrAEs can affect a broad spectrum of organs (Kennedy and Salama, 2020), and the skin and subcutaneous system were most common under ICIs therapy. It is noteworthy that camrelizumab, a PD-1 antibody approved for the treatment of Hodgkin’s lymphoma, hepatocellular carcinoma, non-small cell lung cancer, esophageal cancer, and nasopharyngeal carcinoma by the National Medical Products Administration in China and was designated as an orphan drug by the United States. Food and Drug Administration, has a specific irAE—RCCEP with quite a high incidence ranging from 67% to 79.8% when used alone though the majority of which were grade 1 to 2 (Huang et al., 2020; Qin et al., 2020). Nonetheless, it is reported in phase II trials that camrelizumab combined with apatinib appreciably decreased the risk of RCCEP to 29.5% in advanced hepatocellular carcinoma (Xu et al., 2021) and to 8.9% in advanced cervical cancer patients (Lan et al., 2020). Overall, the toxicity of camrelizumab was acceptable. The mechanism of RCCEP remains unclarified and the VEGFR-2 signaling pathway possibly plays a crucial role in the formation of RCCEP.

### 4.2 Strengths

To our knowledge, this is the first systematic review and meta-analysis that reported the incidence of various types of irAEs of anti-VEGF(R) agents combined with PD-(L)1 inhibitors, which could provide a reference for clinical decision-making. Additionally, we provided evidence of direct and indirect comparisons between PD-(L)1 blockade monotherapy and combined with VEGF(R) blockade therapy, which was more persuasive.

### 4.3 Limitations

There are some limitations in our study to be improved. Predominantly, scarce direct comparisons were included in the meta-analysis. Additionally, publication bias and small-study effects based on funnel plot and Egger’s test were observed in the meta-analyses of some adverse events. What’s more. cancer types, lines of treatment, and duration of follow-up, which may bring in high heterogeneity across several studies, should be taken into consideration. Further, versions of CTCAE were not consistent between the earlier and later conducted studies. Moreover, the incidence of all-cause or treatment-emergent AEs was regarded as the incidence of trAEs when trAEs were not reported or were not reported in detail in the included studies (McDermott et al., 2018; Bonomi et al., 2019; Eng et al., 2019; Levy et al., 2019; Necchi et al., 2019; Pujol et al., 2019; Theelen et al., 2019; Gogas et al., 2021; Jassem et al., 2021; McBride et al., 2021; van der Heijden et al., 2021). There were no trials that either compared different kinds of PD-1 blockade monotherapy (except for drug compared with its biosimilar) or compared different combination regimens, so that we were unable to conduct a network meta-analysis. Risks of irAEs and trAEs differ across different types and dosages of PD-(L)1 antibodies and their combinations with various anti-VEGF(R) drugs, which could be further explored through a network meta-analysis when there are more head-to-head comparisons performed in the future.

## 5 Conclusion

Total incidences of any grade and grade ≥3 irAEs were higher in the combination treatment group. Direct comparisons indicated that no significant difference was observed between the two regimens for any grade and grade ≥3 specific irAEs. RCCEP and thyroid disorders need to be paid attention to clinically. Moreover, there is a need for more trials conducted to directly compare PD-(L)1 inhibitor monotherapy and its combination therapy with anti-VEGF(R) agents. The safety profiles of the two regimens should be further explored. Exploration of the mechanism of action and regulatory management of adverse events should be enhanced.

## Data Availability

The raw data supporting the conclusion of this article will be made available by the authors, without undue reservation.
