# Supplementary material for: Adverse events of PD-(L)1 inhibitors plus anti-VEGF(R) agents compared with PD-(L)1 inhibitors alone for cancer patients: a systematic review and meta-analysis
Source: Front Pharmacol. 2023 Apr 25;14:1093194. doi: 10.3389/fphar.2023.1093194 (PMC10166877; doi:10.3389/fphar.2023.1093194)
Supplement: Supplementary file 1 [file DataSheet1.pdf]

Supplementary Table S1. Search strategy.

PubMed	958 records hit
#1	Programmed Cell Death 1 Receptor[mh] OR PD-1[tiab] OR PD1 Receptor[tiab] OR Receptor, PD1[tiab] OR CD279 Antigen*[tiab] OR Antigen*, CD279[tiab] OR Programmed Cell Death Protein 1[tiab] OR Programmed Cell Death 1 Protein[tiab] OR Pembrolizumab[tiab] OR Nivolumab[tiab] OR Cemiplimab[tiab] OR Toripalimab[tiab] OR Camrelizumab[tiab] OR Tislelizumab[tiab] OR Sintilimab[tiab] OR Penpulimab[tiab] OR Zimberelimab[tiab] OR B7-H1 Antigen[mh] OR Antigen, B7-H1[tiab] OR B7 H1 Antigen[tiab] OR B7-H1 Immune Costimulatory Protein[tiab] OR Antigen*, CD274[tiab] OR CD274 Antigen*[tiab] OR PD-L1[tiab] OR Programmed Cell Death 1 Ligand 1[tiab] OR Programmed Death Ligand 1[tiab] OR Atezolizumab[tiab] OR Durvalumab[tiab] OR Avelumab[tiab]
#2	randomized controlled trial [pt] OR controlled clinical trial [pt] OR randomized [tiab] OR placebo [tiab] OR drug therapy [sh] OR randomly [tiab] OR trial [tiab] OR groups [tiab]
#3	animals[mh] NOT humans[mh]
#4	#2 NOT #3
#5	#1 AND #4
#6	clinical trial [PT] OR randomized controlled trial [PT]
#7	#5 AND #6
#8	Clinical Trial, Phase I[PT] OR Clinical Trial, Phase IV [PT]
#9	#7 NOT #8
**Embase**	**1220 records hit**
#1	'programmed death 1 receptor'/exp OR 'pd-1':ab,ti OR 'pd 1 protein':ab,ti OR 'pd 1 receptor':ab,ti OR 'pd1 receptor':ab,ti OR 'receptor, pd1':ab,ti OR 'programmed cell death protein 1':ab,ti OR 'programmed cell death 1 protein':ab,ti OR 'programmed cell death 1 receptor':ab,ti OR pembrolizumab:ab,ti OR nivolumab:ab,ti OR cemiplimab:ab,ti OR toripalimab:ab,ti OR camrelizumab:ab,ti OR tislelizumab:ab,ti OR sintilimab:ab,ti OR penpulimab:ab,ti OR zimberelimab:ab,ti OR 'programmed death 1 ligand 1'/exp OR 'pd-l1':ab,ti OR 'pd l1 protein':ab,ti OR 'cd274 antigen':ab,ti OR 'programmed death ligand 1':ab,ti OR 'programmed cell death 1 ligand 1':ab,ti OR atezolizumab:ab,ti OR durvalumab:ab,ti OR avelumab:ab,ti
#2	'randomized controlled trial'/exp OR 'controlled clinical trial'/exp OR randomized:ti,ab OR placebo:ti,ab OR 'drug therapy':lnk OR randomly:ti,ab OR trial:ti,ab OR groups:ti,ab
#3	'animal'/exp NOT 'human'/exp
#4	#2 NOT #3
#5	#1 AND #4
#6	[controlled clinical trial]/lim OR [randomized controlled trial]/lim
#7	'phase 1 clinical trial'/de OR 'phase 4 clinical trial'/de
#8	#5 AND #6 NOT #7
#9	[article]/lim OR [article in press]/lim
#10	#8 AND #9
**CENTRAL**	**1860 records hit**
#1	MeSH descriptor: [Programmed Cell Death 1 Receptor] explode all trees
#2	(‘PD-1’):ti,ab,kw OR (‘PD 1 Receptor’):ti,ab,kw OR (‘Programmed Cell Death Protein 1’):ti,ab,kw OR (‘Programmed Cell Death 1 Protein’):ti,ab,kw OR (Pembrolizumab):ti,ab,kw OR (Nivolumab):ti,ab,kw OR (Cemiplimab):ti,ab,kw OR (Toripalimab):ti,ab,kw OR (Camrelizumab):ti,ab,kw OR (Tislelizumab):ti,ab,kw OR (Sintilimab):ti,ab,kw OR (Penpulimab):ti,ab,kw OR (Zimberelimab):ti,ab,kw
#3	MeSH descriptor: [B7-H1 Antigen] explode all trees
#4	(‘Programmed Death Ligand 1’):ti,ab,kw OR (‘Programmed Cell Death 1 Ligand

	1’):ti,ab,kw OR (‘PD-L1’):ti,ab,kw OR (Atezolizumab):ti,ab,kw OR (Durvalumab):ti,ab,kw OR (Avelumab):ti,ab,kw
#5	#1 OR #2 OR #3 OR #4
#6	(Randomized Controlled Trial OR Controlled Clinical Trial):pt
#7	#5 AND #6

Supplementary Table S2. Demographics and main characteristics of studies included in the meta-analysis

Author	Journal	Trial name	NCT	Phase	Masking status	Indication	PD-L1 status	ECOG PS score	Line of treatment	Follow-up (month)	Intervention	Control
Popat 2020	Ann Oncol	PROMISE-meso	NCT02991482	III	Open-label	Pleural mesothelioma	unselected	0-1	≥2	median 11.8 (IQR 9.9-14.5)	Pembrolizumab	Single-agent chemotherapy
Pujol 2019	J Thorac Oncol	IFCT-1603	NCT03059667	II	Open-label	SCLC	unselected	0-2	2	median 13.7 (95% CI 12.7-not reported)	Atezolizumab	Chemotherapy
Singh 2022	Clin Cancer Res	NA	NCT02880020	II	Open-label	Gastrointestinal Stromal Tumors	NA	0-1	≥2	NA	Nivolumab	Nivolumab + ipilimumab
Mahmood 2021	Int J Radiation Oncol Biol Phys	NA	NCT03087019	II	Open-label	ACC	NA	0-1	≥1	median 19.8	Pembrolizumab	Pembrolizumab + hypofractionated radiation therapy
Zhang 2020	Cancer	Keynote 143; RAPID CHECK; ACE-ST-005	NCT02351739	II	Open-label	UC	NA	0-1	≥2	NA	Pembrolizumab	Pembrolizumab + acalabrutinib
Heudobler 2021	Front Pharmacol	ModuLung	NCT02852083	II	Open-label	NSCLC	NA	0-1	≥2	median 8.25	Nivolumab	Biomodulation
Necchi 2019	Eur Urol	APACHE	NCT03081923	II	Open-label	Germ cell tumors	NA	0-1	≥3	median 7.5	Durvalumab	Durvalumab + tremelimumab
Planchard 2020 (Study A)	Ann Oncol	ARCTIC	NCT02352948	III	Open-label	NSCLC	≥25%	0-1	≥3	median 9.1	Durvalumab	SOC
Planchard 2020 (Study B)	Ann Oncol	ARCTIC	NCT02352948	III	Open-label	NSCLC	<25%	0-1	≥3	median 9.1	Durvalumab	Durvalumab + tremelimumab; SOC; Tremelimumab

Finn 2020a	N Engl J Med	Imbrave 150	NCT03 434379	III	Open-label	HCC	NA	0-1	1	median 8.6	Atezolizumab + bevacizumab	Sorafenib
Rini 2019	Lancet	IMmotion151	NCT02 420821	III	Open-label	RCC	unselected	Karnofsky performance score ≥ 70	1	median 24	Atezolizumab + bevacizumab	Sunitinib
van der Heijden 2021	Eur Urol	IMvigor211	NCT02 302807	III	Open-label	UC	unselected	NA	2	median 33 (range 0–42.3)	Atezolizumab	Chemotherapy
Fehrenbacher 2016	Lancet	POPLAR	NCT01 903993	II	Open-label	NSCLC	unselected	0-1	2-3	median 14.8 (range 0.2+ to 19.6)	Atezolizumab	Docetaxel
Galsky 2020	Lancet	IMvigor130	NCT02 807636	III	Double-blind	UC	unselected	0-2	1	median 11.8 (IQR 6.1–17.2)	Atezolizumab	Atezolizumab + platinum-based chemotherapy; Placebo + platinum-based chemotherapy
Eng 2019	Lancet Oncol	IMblaze370	NCT02 788279	III	Open-label	CRC	unselected	0-1	≥3	median 7.3 (IQR 3.7–13.6)	Atezolizumab	Atezolizumab + cobimetinib; Regorafenib
Pujade-Lauraine 2021	Lancet Oncol	JAVELIN Ovarian 200	NCT02 580058	III	Open-label	Ovarian cancer	unselected	0-1	2-4[a]	median 18.2 (15.8–21.2)	Avelumab	Avelumab + PLD; PLD
Motzer 2019	N Engl J Med	JAVELIN Renal 101	NCT02 684006	III	Open-label	RCC	unselected	0-1	1	median 11.6	Avelumab + Axitinib	Sunitinib
Park 2021	J Thorac Oncol	JAVELIN Lung 200	NCT02 395172	III	Open-label	NSCLC	unselected	0-1	2	2-year	Avelumab	Docetaxel
Huang 2020	Lancet	ESCORT	NCT03	III	Open-	ESCC	unselected	0-1	2	median 8.3	Camrelizuma	Chemotherapy

	Oncol		099382		label					(IQR 4.1–12.8)	b	
Sezer 2021	Lancet	EMPOWER-Lung 1	NCT03 088540	III	Open-label	NSCLC	≥50%	0-1	1	median 10.8 (IQR 7.6–15.8)	Cemiplimab	Chemotherapy
Bonomi 2019	Oncol Lett	NA	NCT02 581943	II	Open-label	NSCLC	NA	2-3	2	NA	Pembrolizum ab	Pembrolizumab + carboplatin + paclitaxel
McDermott 2018	Nat Med	IMmotion150	NCT01 984242	II	Open-label	RCC	unselected	Karnofs ky performa nce score ≥ 70	1	median 20.7	**Atezolizuma b + bevacizumab**	**Atezolizumab; Sunitinib**
Gogas 2021	Ann Oncol	IMspire170	NCT03 273153	III	Open-label	Melanoma	unselected	0-1	1	median 7.2 (IQR 4.9-10.1)	Pembrolizum ab	Cobimetinib + atezolizumab
Long 2018	Lancet Oncol	NA	NCT02 374242	II	Open-label	Melanoma brain metastases	unselected	0-2	1	median 17 (IQR 8–25)	Nivolumab	Nivolumab + ipilimumab
Powles 2020a	Lancet Oncol	DANUBE	NCT02 516241	III	Open-label	UC	unselected	0-1	1	median 41.2 (IQR 37.9–43.2)	Durvalumab	Durvalumab + tremelimumab; Chemotherapy
O'Reilly 2019	JAMA Oncol	NA	NCT02 558894	II	Open-label	PDAC	unselected	0-1	2	median 3.2 (range 0.4-18.1)	Durvalumab	Durvalumab + tremelimumab
Ferris 2020	Ann Oncol	EAGLE	NCT02 369874	III	Open-label	HNSCC	unselected	0-1	2	median 7.6	Durvalumab	Durvalumab + tremelimumab; SOC
Rizvi 2020	JAMA Oncol	MYSTIC	NCT02 453282	III	Open-label	NSCLC	unselected	0-1	1	median 30.2 (range 0.3-37.2)	Durvalumab	Durvalumab + tremelimumab; Chemotherapy
Reardon 2020	JAMA Oncol	CheckMate 143	NCT02 017717	III	Open-label	Glioblastoma	unselected	Karnofs ky performa nce	2	median 9.5	Nivolumab	Bevacizumab

								score ≥ 70				
Theelen 2019	JAMA Oncol	PEMBRO-RT	NCT02492568	II	Open-label	NSCLC	unselected	0-1	≥2	median 23.6 (range 0.1-34.4)	Pembrolizumab	Pembrolizumab after radiotherapy
Shitara 2020	JAMA Oncol	KEYNOTE-062	NCT02494583	III	Partially blinded[c]	GC/GEJC	PD-L1 CPS ≥ 1	0-1	1	median 29.4 (range 22.0-41.3)	Pembrolizumab	Pembrolizumab + chemotherapy; Chemotherapy
Long 2019	Lancet Oncol	KEYNOTE-252/ECHO-301	NCT02752074	III	Double-blind	Melanoma	unselected	0-1	1[b]	median 12.4 (IQR 10.3–14.5)	Placebo + pembrolizumab	Epacadostat + pembrolizumab
Hamid 2017	Eur J Cancer	KEYNOTE-002	NCT01704287	II	Partially blinded[d]	Melanoma	unselected	0-1	≥1	median 28 (range 24.1-35.5)	Pembrolizumab 2 mg/kg; Pembrolizumab 10 mg/kg	Chemotherapy
Carbone 2017	N Engl J Med	CheckMate 026	NCT02041533	III	Open-label	NSCLC	≥1%	0-1	1	minimum 13.7, median 13.5[e]	Nivolumab	Chemotherapy
Robert 2020	J Clin Oncol	CheckMate 066	NCT01721772	III	Double-blind	Melanoma	unselected	0-1	1	minimum 60, median 32.0	Nivolumab	Dacarbazine
Reck 2021	J Clin Oncol	KEYNOTE-024	NCT02142738	III	Open-label	NSCLC	PD-L1 TPS ≥ 50%	0-1	1	5-year	Pembrolizumab	Chemotherapy
Larkin 2019	N Engl J Med	CheckMate 067	NCT01844505	III	Quadruple-blind	Melanoma	unselected	0-1	1	minimum 60, median 36.0	Nivolumab	Nivolumab + ipilimumab; Ipilimumab
Liu 2021	J Immunother Cancer	NA	NCT02961101; NCT03250962	II	Open-label	cHL	unselected	0-1	≥3	median 34.5 (range 28.4–42.3)	Camrelizumab	Decitabine + camrelizumab

Motzer 2021	N Engl J Med	CLEAR	NCT02811861	III	Open-label	RCC	unselected	Karnofsky performance score ≥ 70	1	median 26.6	Lenvatinib + pembrolizumab	Lenvatinib + everolimus; Sunitinib
Herbst 2020	J Clin Oncol	KEYNOTE-010	NCT01905657	II/III	Open-label	NSCLC	PD-L1 expression on at least 1% of tumour cells (ie, a TPS ≥ 1%)	0-1	≥2	median 42.6 (range 35.2-53.2)	Pembrolizumab	Docetaxel
Boku 2021	Gastric Cancer	ATTRACTION-2	NCT02267343	III	Double-blind	Gastric cancer	unselected	0-1	≥3	median 38.5 (range 36.1–47.5)	Nivolumab	Placebo
Scherpereel 2019	Lancet Oncol	IFCT-1501 MAPS2	NCT02716272	II	Open-label	MPM	unselected	0-1	2-3	median 20.1 (IQR 19.6–20.3)	Nivolumab	Nivolumab + ipilimumab
Choueiri 2021	N Engl J Med	CheckMate 9ER	NCT03141177	III	Open-label	RCC	unselected	Karnofsky performance score ≥ 70	1	median 18.1 (range 10.6-30.6)	Nivolumab + cabozantinib	Sunitinib
Hellmann 2019	N Engl J Med	CheckMate 227	NCT02477826	III	Open-label	NSCLC	≥1%	0-1	1	minimum 28.3	Nivolumab	Nivolumab + ipilimumab; Chemotherapy

Gettinger 2021	JAMA Oncol	Lung-MAP S1400I	NCT02785952	III	Open-label	NSCLC	unselected	Zubrod performance status score 0-1	≥2	median 29.5 (95%CI 26.0-32.8) in surviving patients	Nivolumab	Nivolumab + ipilimumab
Kato 2019	Lancet Oncol	ATTRACTION-3	NCT02569242	III	Open-label	ESCC	unselected	0-1	2	minimum 17.6	Nivolumab	Chemotherapy
Lu 2021	Lung Cancer	CheckMate 078	NCT02613507	III	Open-label	NSCLC	unselected	0-1	2	minimum 25.9	Nivolumab	Docetaxel
Borghaei 2015	N Engl J Med	CheckMate 057	NCT01673867	III	Open-label	nonsquamous NSCLC	unselected	0-1	2-3	minimum 13.2	Nivolumab	Docetaxel
Brahmer 2015	N Engl J Med	CheckMate 017	NCT01642004	III	Open-label	squamous-cell NSCLC	unselected	0-1	2	minimum approximately 11	Nivolumab	Docetaxel
Motzer 2020	Cancer	CheckMate 025	NCT01668784	III	Open-label	RCC	unselected	Karnofsky performance score ≥ 70	2-3	minimum 64, median 72	Nivolumab	Everolimus
Hamanishi 2021	J Clin Oncol	NINJA	NA	III	Open-label	Ovarian Cancer	unselected	0-1	≥2	NA	Nivolumab	Chemotherapy (Gemcitabine or PLD)
Ferris 2018	Oral Oncol	CheckMate 141	NCT02105636	III	Open-label	HNSCC	unselected	0-1	≥2	minimum 24.2	Nivolumab	Investigator's choice
D'Angelo 2018	Lancet Oncol	Alliance A091401	NCT02500797	II	Open-label	Sarcoma	NA	0-1	≥2	median 13.6 (IQR 8.9–15.88)	Nivolumab	Nivolumab + ipilimumab
Larkin 2018	J Clin Oncol	CheckMate 037	NCT01721746	III	Open-Label	Melanoma	unselected	0-1	≥2	approximately 2 years	Nivolumab	Investigator's choice chemotherapy

Powles 2021	Lancet Oncol	KEYNOTE-361	NCT02853305	III	Open-label	UC	unselected	0-2	1	median 31.7 (IQR 27.7–36.0)	Pembrolizumab	Pembrolizumab + chemotherapy; Chemotherapy
Burtness 2019	Lancet	KEYNOTE-048	NCT02358031	III	Open-label	HNSCC	unselected	0-1	1	median 11.5 (IQR 5.1−25.7)	Pembrolizumab	Pembrolizumab + chemotherapy; Cetuximab + chemotherapy
Finn 2020b	J Clin Oncol	KEYNOTE-240	NCT02702401	III	Double-blind	HCC	NA	0-1	2	median 13.8 (range 0.9-30.4)	Pembrolizumab	Placebo
André 2020	N Engl J Med	KEYNOTE-177	NCT02563002	III	Open-label	MSI-H–dMMR CRC	NA	0-1	1	median 32.4 (range 24.0-48.3)	Pembrolizumab	Chemotherapy
Powles 2020b	Lancet Oncol	KEYNOTE-426	NCT02853331	III	Open-label	RCC	unselected	Karnofsky performance score ≥ 70	1	median 30.6 (IQR 27.2–34.2; range 23.4–38.4)	Pembrolizumab + axitinib	Sunitinib
Boyer 2021	J Clin Oncol	KEYNOTE-598	NCT03302234	III	Double-blind	NSCLC	PD-L1 TPS ≥ 50%	0-1	1	median 20.6 (range 12.4-31.7)	Pembrolizumab + placebo	Pembrolizumab + ipilimumab
Kuruvilla 2021	Lancet Oncol	KEYNOTE-204	NCT02684292	III	Open-label	cHL	unselected	0-1	≥2	median 25.7 (IQR 23.4–33.0)[f]	Pembrolizumab	Brentuximab vedotin
Mok 2019	Lancet	KEYNOTE-042	NCT02220894	III	Open-label	NSCLC	PD-L1 TPS ≥ 1%	0-1	1	median 12.8 (IQR 6.0–20.0)	Pembrolizumab	Chemotherapy
Winer 2021	Lancet Oncol	KEYNOTE-119	NCT02555657	III	Open-label	TNBC	unselected	0-1	2-3	median 31.4 (IQR 27.8–34.4)	Pembrolizumab	Chemotherapy

Robert 2019	Lancet Oncol	KEYNOTE-006	NCT01 866319	III	Open-label	Melanoma	unselected	0-1	1-2	median 57.7 (IQR 56.7–59.2)	Pembrolizumab	Ipilimumab
Cohen 2019	Lancet	KEYNOTE-040	NCT02 252042	III	Open-label	HNSCC	unselected	0-1	≥2	median 8.4 (3.3–14.5)	Pembrolizumab	SOC
Fuchs 2022	Gastric Cancer	KEYNOTE-061	NCT02 370498	III	Open-label	GC/GEJC	PD-L1 CPS ≥ 1	0-1	2	4 years and 4 months	Pembrolizumab	Paclitaxel
Chawla 2022	J Clin Oncol	IMDZ-C232	NCT02 609984	II	Open-label	STS	NA	0-1	≥2	NA	Atezolizumab	CMB305 + atezolizumab
Bang 2018	Ann Oncol	JAVELIN Gastric 300	NCT02 625623	III	Open-label	GC/GEJC	unselected	0-1	3	median 10.6 (range 0.1–17.8)	Avelumab	Chemotherapy
Levy 2019	Eur J Cancer	NA	NCT02 546986	II	Double-blind	NSCLC	unselected	0-1	2	median 12.2	Pembrolizumab + placebo	Pembrolizumab + CC-486 (oral azacitidine)
Nayak 2021	Clin Cancer Res	NA	NCT02 337491	II	Open-label	Glioblastoma	unselected	Karnofsky performance score ≥ 70	2-3	median 48.6 (95%CI, 48.6–not reached) for pembrolizumab with bevacizumab; median 49.4 (95% CI, 48.6–not reached) for pembrolizumab	**Pembrolizumab + bevacizumab**	**Pembrolizumab**
Zamarin 2020	J Clin Oncol	NRG GY003	NCT02 498600	II	Open-label	Ovarian Cancer	unselected	0-2	2-4	first-stage: median approximately 33 second-stage: median	Nivolumab	Nivolumab + ipilimumab

										approximately 11		
McBride 2021	J Clin Oncol	NA	NCT02684253	II	Open-label	HNSCC	unselected	0-2	≥1	median 20.2 in the surviving group	Nivolumab	Nivolumab + SBRT
Fradet 2019	Ann Oncol	KEYNOTE-045	NCT02256436	III	Open-label	UC	unselected	0-2	2	median 27.7	Pembrolizumab	Chemotherapy
Kojima 2020	J Clin Oncol	KEYNOTE-181	NCT02564263	III	Open-label	Esophageal cancer	unselected	0-1	2	median 7.1 (range 0.5-31.3)	Pembrolizumab	Chemotherapy
Siu 2019	JAMA Oncol	CONDOR	NCT02319044	II	Open-label	HNSCC	PD-L1–low/negative[g]	0-1	2	median 6.0 (range 0.3-18.0)	Durvalumab	Durvalumab + tremelimumab; Tremelimumab
Spigel 2021	Ann Oncol	CheckMate 331	NCT02481830	III	Open-label	SCLC	unselected	0-1	2	median 7.0	Nivolumab	Chemotherapy
Fehrenbacher 2018	J Thorac Oncol	OAK	NCT02008227	III	Open-label	NSCLC	unselected	0-1	2-3	median 26, minimum 21	Atezolizumab	Docetaxel
Jassem 2021	J Thorac Oncol	IMpower110	NCT02409342	III	Open-label	NSCLC	≥1%	0-1	1	median 31.3 (range 0–52), 31.0 (range 0–52), 30.0 (range 0–52) in the high, high-or-intermediate, and any PD-L1 expression wild type groups	Atezolizumab	Platinum-based chemotherapy

Abbreviations: NA: Not applicable; UC: Urothelial cancer; NSCLC: Non-small cell lung cancer; ACC: Adenoid cystic carcinoma; RCC: Renal cell carcinoma; HNSCC: Head and neck squamous cell carcinoma; HCC: Hepatocellular carcinoma; CRC: Colorectal cancer; ESCC: Esophageal squamous cell carcinoma; PDAC: Pancreatic ductal adenocarcinoma; GC/GEJC: Gastric cancer/gastroesophageal junction cancer; cHL: classical Hodgkin lymphoma; MPM: Malignant pleural mesothelioma; TNBC: Triple-negative breast cancer; STS: Soft-tissue sarcomas; ECOG PS score: Eastern Cooperative Oncology Group Performance status score; SOC: Standard of

care; PLD: Pegylated liposomal doxorubicin

Bold indicated the direct comparisons of PD-(L)1 inhibitors combined with VEGF(R) blockades with PD-(L)1 inhibitors monotherapy.

Note:

a: A maximum of three previous lines for platinum-sensitive disease (most recent line containing platinum) with no previous systemic therapy for platinum-resistant disease.

b: BRAF directed therapy was eligible.

c: Patients and site and sponsor personnel were blinded to pembrolizumab or placebo in the combination therapy groups. Investigators and patients were unblinded to pembrolizumab monotherapy.

d: Patients and investigators masked only to the pembrolizumab dose.

e: The minimum follow-up was computed as the time from randomization of the last patient to the database lock, and the median follow-up was computed for all the patients from randomization to the last known vital-status date.

f: Median time from randomisation to data cutoff.

g: TC<25%, using the validated VENTANA PD-L1 [SP263] Assay [Ventana Medical Systems]

Supplementary Table S3. Methodological quality of studies eligible.

Study	Randomization	Allocation concealment	Blinding of participants and staff	Blinding of outcome assessors	Incomplete outcome data	Selective outcome reporting	Other sources of bias
Popat 2020	Low	Low	High	Low	Low	Low	Low
Pujol 2019	Low	Low	High	Low	Low	Low	Low
Singh 2022	Low	Low	High	High	Low	Low	Low
Mahmood 2021	Low	Low	High	High	Low	Low	Low
Zhang 2020	Low	Low	High	High	Low	Low	Low
Heudobler 2021	Low	Low	High	High	Low	Low	Low
Necchi 2019	Low	Low	High	High	Low	Low	Low
Planchard 2020 (Study A)	Low	Low	High	High	Low	Low	Low
Planchard 2020 (Study B)	Low	Low	High	High	Low	Low	Low
Finn 2020a	Low	Low	High	High	Low	Low	Low
Rini 2019	Low	Low	High	Low	Low	Low	Low
van der Heijden 2021	Low	Low	High	Low	Low	Low	Low
Fehrenbacher 2016	Low	Low	High	High	Low	Low	Low
Galsky 2020	Low	Low	High	High	Low	Low	Low
Eng 2019	Low	Low	High	High	High	Low	Low
Pujade-Lauraine 2021	Low	Low	High	High	Low	Low	Low
Motzer 2019	Low	Low	High	High	Low	High	Low
Park 2021	Low	Low	High	High	Low	Low	Low
Huang 2020	Low	Low	High	High	Low	Low	Low
Sezer 2021	Low	Low	High	High	Low	Low	Low

Bonomi 2019	Low	Low	High	High	Low	Low	Low
McDermott 2018	Low	Low	High	High	Low	Low	Low
Gogas 2021	Low	Low	High	High	Low	Low	Low
Long 2018	Low	Low	High	High	Low	Low	Low
Powles 2020a	Low	Low	High	High	Low	Low	Low
O'Reilly 2019	Low	Low	High	High	Low	Low	Low
Ferris 2020	Low	Low	High	High	Low	Low	Low
Rizvi 2020	Low	Low	High	High	Low	Low	Low
Reardon 2020	Low	Low	High	High	High	Low	Low
Theelen 2019	Low	Low	High	High	Low	Low	Low
Shitara 2020	Low	Low	High	High	Low	Low	Low
Long 2019	Low	Low	Low	Low	Low	Low	Low
Hamid 2017	Low	Low	High	High	Low	Low	Low
Carbone 2017	Low	Low	High	High	Low	Low	Low
Robert 2020	Low	Low	Low	Low	Low	High	Low
Reck 2021	Low	Low	High	High	Low	Low	Low
Larkin 2019	Low	Low	Low	Low	Low	Low	Low
Liu 2021	Low	Low	High	High	Low	Low	Low
Motzer 2021	Low	Low	High	High	Low	Low	Low
Herbst 2020	Low	Low	High	High	Low	Low	Low
Boku 2021	Low	Low	Low	Low	Low	Low	Low
Scherpereel 2019	Low	Low	High	High	Low	Low	Low
Choueiri 2021	Low	Low	High	High	Low	Low	Low
Hellmann 2019	Low	Low	High	High	Low	Low	Low
Gettinger 2021	Low	Low	High	High	Low	Low	Low

Kato 2019	Low	Low	High	High	Low	Low	Low
Lu 2021	Low	Low	High	High	Low	Low	Low
Borghaei 2015	Low	Low	High	High	Low	Low	Low
Brahmer 2015	Low	Low	High	High	Low	Low	Low
Motzer 2020	Low	Low	High	High	Low	Low	Low
Hamanishi 2021	Low	Low	High	High	Low	Low	Low
Ferris 2018	Low	Low	High	High	Low	Low	Low
D'Angelo 2018	Low	Low	High	High	Low	Low	Low
Larkin 2018	Low	Low	High	High	High	Low	Low
Powles 2021	Low	Low	High	High	Low	Low	Low
Burtness 2019	Low	Low	High	High	Low	Low	Low
Finn 2020b	Low	Low	Low	Low	Low	Low	Low
André 2020	Low	Low	High	High	Low	Low	Low
Powles 2020b	Low	Low	High	High	Low	Low	Low
Boyer 2021	Low	Low	Low	Low	Low	Low	Low
Kuruvilla 2021	Low	Low	High	High	Low	Low	Low
Mok 2019	Low	Low	High	High	Low	Low	Low
Winer 2021	Low	Low	High	High	Low	Low	Low
Robert 2019	Low	Low	High	High	Low	Low	Low
Cohen 2019	Low	Low	High	High	Low	Low	Low
Fuchs 2022	Low	Low	High	High	Low	Low	Low
Chawla 2022	Low	Low	High	High	Low	Low	Low
Bang 2018	Low	Low	High	High	Low	Low	Low
Levy 2019	Low	Low	Low	Low	Low	Low	Low
Nayak 2021	Low	Low	High	High	Low	Low	Low

Zamarin 2020	Low	Low	High	High	Low	Low	Low
McBride 2021	Low	Low	High	High	Low	Low	Low
Fradet 2019	Low	Low	High	High	Low	High	Low
Kojima 2020	Low	Low	High	High	Low	Low	Low
Siu 2019	Low	Low	High	High	Low	Low	Low
Spigel 2021	Low	Low	High	High	Low	Low	Low
Fehrenbacher 2018	Low	Low	High	High	Low	Low	Low
Jassem 2021	Low	Low	High	High	Low	Low	Low
